# Clinical teachers as positive and negative role models: an explanatory sequential mixed method design

**DOI:** 10.18502/jmehm.v12i11.1448

**Published:** 2019-09-04

**Authors:** Leila Bazrafkan, Ali Asghar Hayat, Seyed Ziaaddin Tabei, Leila Amirsalari

**Affiliations:** 1 *Assistant Professor, Clinical Education Research Center, Shiraz University of Medical Sciences, Shiraz, Iran.*; 2 *Professor, Department of Medical Ethics and Philosophy in Health Care, School of Medicine, Shiraz University of Medical Sciences, Shiraz, Iran.*; 3 *Researcher, Department of Pathology, School of Medicine, Shiraz University of Medical Sciences, Shiraz, Iran. *

**Keywords:** Role model, Clinical teacher, Medical education, Medical students

## Abstract

Today, role modeling is an essential component of medical education that facilitates the students' learning and affects their attitudes and behaviors. Hence, this study aimed to examine the characteristics of positive and negative role models using a mixed method approach. In the quantitative part, data were collected using a questionnaire with 24 items. The research population included medical students who were in their clinical period between May 2017 and December 2018 at Shiraz University of Medical Sciences (n = 750). A total of 282 questionnaires were completed by these students, and in the qualitative part, 26 semi-structured interviews were conducted with them.

The most important components of role modeling for students included: individual characteristics, clinical skills and competence, teaching skills and professionalism, in that order. The qualitative analysis confirmed the results of the quantitative analysis. The findings showed that the characteristics of a negative role model can also be classified in four main components. The results demonstrated that 46.8% of the students identified one or more medical teachers as negative models.

Students paid attention to not only the positive characteristics of their teachers, but also their negative features, stating that they had been influenced by both. Therefore, it can be concluded that clinical teachers should improve their performance as positive role models through reducing these negative effects and reinforcing positive characteristics.

## Introduction

Today, role modeling is an integral and essential component of medical education (1 - 5). Role models facilitate medical students' learning and affect their attitudes, behaviors and ethics (2 - 4, 6 - 9). Moreover, they form the students' values and professional identity and influence their behavior regarding provision of care services. In other words, students' professional identity develops by observing their teachers communicate and interact with patients, colleagues and others (2, 7, 10 - 13). In this regard, some researchers believe that training by way of modeling is one of the most effective methods to transfer experiences and improve students’ professional attitudes (6, 14). It helps medical students to develop high standards of professionalism and affects their career choices and satisfaction (2, 15). Students learn through observing their teachers and emulating their behavior not only in clinical and formal settings, but also in other situations (4). Therefore, teachers should understand that their interactions and behaviors could impact students (1, 2, 7, 16) both in a positive and negative manner (2, 7, 16). Students directly observe their teachers’ positive and negative behaviors. In this regard, Wright discovered that more than 50 percent of the faculty members were not perceived as positive role models by the residents (17). Medical students often saw a discrepancy between what they had learnt in the classroom and what they observed in other settings (18). Therefore, clinical teachers should always be conscious that all their interactions, personal views, behaviors and attitudes are observed and followed by students under the circumstances (18). 

Previous studies on role modeling showed that personality traits, teaching skills and clinical competence are the key components for medical students in selecting role models (2, 4, 5, 19). In a study by Wright and Carrese (2), the results showed that certain features of role modeling pertained to several domains such as personal characteristics (interpersonal skills, positive attitude, commitment to excellence, growth, integrity and leadership), and teaching behaviors (establishing rapport with learners, development and promotion of specific teaching methods and philosophies, and commitment to improving learners). Moreover, perceived barriers to an effective role model comprised being impatient, stubborn and reserved; going too far; and having difficulty remembering names and faces (2). 

The results of another study by Wright et al. (1) showed that the most important factors in selecting role models were personality, clinical skills and competence, and the ability to teach, while research achievements and academic position were the least important. The study also showed that 90 percent of the students had selected a teacher as a role model while they were in medical school (1). 

Several studies have been conducted on characteristics of role models, but most of them have been sporadically carried out in Western countries using a quantitative or qualitative approach, and may not have covered all aspects of role models. In Iran, there has been little research on role modeling (20); thus, given the significant effect of role models in all areas of clinical education and their efficacious impact on personality formation, identity and professional behavior of medical students, this study was an attempt to fill this gap using a mixed method design.

## Methods

This study was conducted using an explanatory sequential mixed method design, which includes a two-phase scheme. In the first phase, the researcher gathers quantitative data and analyzes the findings, and in the second, uses the results to design (or build onto) the qualitative part. The general purpose of this project was to collect qualitative data that would help explain in more detail the initial quantitative results and explore them in more depth (21). 

***The Quantitative Phase of the Study***

Participants were medical students from Shiraz University of Medical Sciences in their externship or internship periods in the years 2017 - 2018 (n = 750). They were selected from among the students in the hospital and dormitories using convenience sampling. In this section, we first developed a 27-item questionnaire by reviewing the literature and background related to role model components identified in previous studies (2, 5, 12, 22 - 24). Content and face validity were used to examine the validity of the questionnaire, and three questions that did not meet the criteria were subsequently eliminated. Second, Kaiser-Meyer-Olkin (KMO) and Bartlett’s Test of Sphericity indicated that the use of factor analysis and classification of questions were permissible (Bartlett = 1602.95; *P*-value ≤ 0.01). The results of the KMO Test (0.71) showed that research data were suitable for factor analysis. Finally, the results obtained by factor analysis (principal components analysis) with Varimax rotation confirmed that there were four factors in the items, which were then classified into four groups: individual characteristics, teaching skills, clinical skills and competence, and professionalism.

Internal consistency reliability was assessed through composite reliability (CR) and Cronbach's alpha, and the values were over the recommended criterion of 0.7 for all constructs. To determine the convergent validity, factor loadings and average variance extracted (AVE) were assessed. If all item loadings are greater than 0.7, they can be considered appropriate (25), and according to the findings, all the items indicated a loading higher than 0.7 on their corresponding construct with appropriate AVE ranging from 0.51 to 0.68. Therefore, reliability and validity of the research constructs were attained. The students were then asked to give their views about each item in a 5-point Likert-scale questionnaire. In total, 282 questionnaires were completed and returned by the participants. The data were analyzed using SPSS version 16 and Smart PLS2, and descriptive statistics (Mean and SD) and inferential statistics (one-way ANOVA and independent t-test) were also applied. 

***The Qualitative Phase of the Study***

In the second stage, we used a qualitative research approach with purposeful sampling to explore medical students’ perceptions and experiences of role modeling in their clinical teachers through semi-structured interviews. After explaining the aims of the study, we asked the participants to answer the following questions: “Is there a teacher in Shiraz University of Medical Sciences who has positive role model characteristics?”; “Describe the characteristics of a teacher who is a positive role model.”; “Is there a teacher in Shiraz University of Medical Sciences who has negative role model characteristics? (Anonymous)”; and “Describe the characteristics of a teacher who is a negative role model (Anonymous)”. We conducted a total of 26 interviews with medical students. It is important to state that data were saturated in the 22^nd^ interview when no further new data were obtained, but we continued to the 26^th^ interview. Each interview lasted 25 to 40 minutes. All interviews were recorded and then typed. Qualitative content analysis was used for data analysis after adjusting the notes obtained from the interviews. First, we read and reviewed all the texts line by line repeatedly to achieve immersion and obtain an overall understanding of the subject. Then, we extracted the initial codes and continued the process. The extracted codes were reread frequently and sorted into subcategories based on their similarities. Next, we compared the subcategories with one another and placed them in broader categories based on their similar characteristics. To ensure reliability and validity, we used four criteria: credibility, transferability, dependability and confirmability, introduced by Guba and Lincoln in the 1980s (26). In addition, to ensure qualitative rigor, we used member checks when coding as well as peer debriefing, and confirmed the results with the participants. For this study, we obtained permission from the Research Ethics Committee of Shiraz University of Medical Sciences (IR.Sums.Rec.1387. S3967). However, anonymity of the students was provided using ID codes, written informed consent was obtained from each participant, and confidentiality of the identities of both students and teachers was considered.

## Results

***The Quantitative Phase of the Study***

The response rate in this study was 75.6%. After collecting the questionnaires and eliminating the low validity ones, 282 questionnaires were analyzed. Descriptive analysis showed that the students’ mean age was 23 3.37, and 39.4% were students, 35.8% externs, and 24.8% interns. In terms of gender, 33.7% were male and 66.3% female. 

Based on the obtained information, the most important component of role modeling for students was individual characteristics, followed by clinical skills and competence, teaching skills, and finally professionalism. From the male students' perspective, the most important component was clinical skills and competence and the least important one was professionalism. According to the female students, the most important component was individual characteristics and the least important was professionalism (Table 1).

**Table1:** The most important components of role modeling from medical students’ point of view

Rank	Student (n=111) Mean & SD	Extern (n=101) Mean & SD	Intern (n=70) Mean & SD	Male (n=95) Mean & SD	Female (n=187) Mean & SD	Total (n=282) Mean & SD
R 1	Individual characteristics (4.48± 0.053)	Individual characteristics (4.46± 0.51)	Clinical skills & competence (4.45± 1.22)	Clinical skills & competence (4.11± 0.65)	Individual characteristics (4.51± 0.50)	Individual characteristics (4.38± 0.58)
R 2	Teaching skills (4.25± 0.56)	Teaching skills (4.26± 0.52)	Individual characteristics (4.11± 0.68)	Individual characteristics (4.38± 0.62)	Teaching skills (4.28± 0.52)	Clinical skills & competence (4.22± 0.76
R 3	Clinical skills & competence (4.16± 0.51)	Clinical skills & competence (4.14± 0.53	Teaching skills (3.94± 0.66)	Teaching skills (3.96± 0.65)	Clinical skills & competence (4.19± 0.82	Teaching skills (4.18± 0.59)
R 4	Professionalism (3.87± 0.68)	Professionalism (3.91± 0.62)	Professionalism (3.66± 0.57)	Professionalism (3.74± 0.72)	Professionalism (3.88± 0.60)	Professionalism (3.83± 0.64)

*SD= standard deviation*

The ANOVA test showed a significant difference in all four components of role modeling based on the students’ educational level (*P *< 0.01). Consequently, Scheffe’s post hoc test was used to investigate the difference between the groups of students (based on educational level) in terms of role model components (Table 2).

**Table 2:** Comparison of students’ views at different levels regarding role model components

**ANOVA**							
		**SS**	**df**	**MS**	**F**	**P- value**	**Post hoc**
**Individual Characteristics**	Between Groups	6.90	2	3.452	10.60	0.001	Stu > Ext
	Within Groups	90.85	279	0.326			Stu > Int*
	Total	97.76	281				Ext > Int*
**Teaching Skills**	Between Groups	5.20	2	2.603	7.78	0.001	Stu < Ext
	Within Groups	93.22	279	0.334			Stu > Int*
	Total	98.42	281				Ext > Int*
**Clinical Skill & Competence**	Between Groups	4.82	2	2.411	4.18	0.016	Stu > Ext
	Within Groups	160.87	279	0.576			Stu < Int*
	Total	165.64	281				Ext < Int*
**Professionalism**	Between Groups	2.927	2	1.464	3.55	0.030	Stu < Ext
	Within Groups	114.90	279	0.412			Stu > Int
	Total	117.83	281				Ext > Int*

*Stu: student; Ext: extern; Int: intern; SS=Sum of Squares; df= Degree of freedom; MS= Mean Square*

* *. The mean difference is significant at the 0.05 level*

Finally, the independent t-test showed that there was a significant difference in the two components of clinical skills and competence, and professionalism between male and female students’ views (Table 3).

**Table 3:** Comparison of male and female students’ views regarding role model components

	Sex	Mean	SD	Mean Difference	T-Test	*P*-value
Individual Characteristics	Male	4.11	0.65	-0.40	-5.37	0.001
	Female	4.51	0.50			
Teaching Skills	Male	3.96	0.65	-0.31	-4.39	0.001
	Female	4.28	0.52			
Clinical Skills & Competence	Male	4.28	0.62	0.08	0.91	0.36
	Female	4.19	0.82			
Professionalism	Male	3.74	0.72	-0.14	-1.73	0.08
	Female	3.88	0.60			

*SD= standard deviation*

***The Qualitative Analysis ***

In general, the results of the participants' responses and the coding process showed that the characteristics of teachers who were positive role models were grouped in 5 main categories and 43 subcategories (Table 4).

**Table 4:** The characteristics of teachers who can be positive role models based on medical students’ views

Themes	Subthemes
Professionalism	Respect for and empathy with patients and students, respect for the rights of coworkers, appropriate behaviors, commitment to professional development, and accepting criticism.
Clinical Skills and Competence	Performing detailed examinations, sound clinical judgment and reasoning, good decision-making abilities, competence in patient management, diagnostic skills, the ability to manage rounds, cooperation, the capacity to monitor all aspects of treatment, and effective communication.
Teaching Skills	Excellent and interactive teaching, employing new teaching methods, providing proper feedback, encouraging student participation in education.
Updated Knowledge and Information	Professional mastery, high academic achievements, updated information, using the power of knowledge, conducting valuable research.
Individual Traits	Confidence, intelligence, the power to convey knowledge, piety and modesty, tireless, caring, ready to help others, committed and patient, good manners, punctuality, authority, the ability to adjust to the team, conscientiousness, sociability, creativity, and honesty.

*1) Professionalism*

The findings suggested that the students considered teachers with higher levels of professionalism as positive role models. In this regard, a participant pointed out, *“A professor may set the students an example of treating patients and students with respect”. *Another participant argued,* “A professor should possess a professional character and observe good manners”.*

*2) Individual Characteristics*

Our findings showed that the students considered personal characteristics among the most important features of positive role models. One participant stated, *“A doctor should treat their audience gently and be kind to them”*. Another student said, *“I believe a professor should be kind and gentle”. *

*3) Teaching Skills*

The qualitative study results showed that from the students’ point of view, teaching skills were among the components of positive role models. In this regard, a participant stated, *“Not only is a professor expected to exhibit professional behavior, but also to deliver quality teaching, interact with students and provide what they need”*. Another participant said, *“Once a professor shows up in class, you have the feeling that she/he is doing her/his best to educate the students to accept responsibility. She/he would want to teach all that she/he has learned”*.

*4) Clinical Skills and Competence*

Our findings showed that the students considered clinical competence as an important feature of positive role models. One of the students stated, *“To me, a good clinical professor is a good physician who has sound clinical judgment and reasoning”*. 

*5) Updated Knowledge and Information*

The results revealed that the students were specifically concerned with the lecturers’ knowledge and scientific competency. In this respect, one of the students said, *“In my opinion, a lecturer should have sufficient knowledge and expertise as well as good conduct and manners”*.

Considering the question*” Is there a teacher in Shiraz University of Medical Sciences who has positive role model characteristics”*, the obtained result showed that 79 percent of the students acknowledged one or more teachers as role models, while about 6 percent explicitly stated that there was no positive role model at their university, and finally about 15% did not answer the question (Table 5).

**Table 5:** Students’ answers about positive role models at the university

Question 2	Positive Answers	Negative Answers	No Answers
Is there a teacher at the Shiraz University of Medical Sciences who has positive role model characteristics?	222 (79%)	17 (6%)	43 (15%)

As to the question *“Describe the characteristics of a teacher who is a negative role model (Anonymous)”*, the results obtained from the coding process showed that, based on the students' views, characteristics of a teacher who is a negative role model could be grouped in 4 main categories and 28 subcategories (Table 6). 

**Table 6:** Characteristics of negative role models based on students' views

Themes	Subthemes
Lack of Professional Behaviors	Bad behavior toward students, failure to respect the dignity of others, bad behavior toward patients and their relatives, regarding students as competitors, criticizing other teachers, looking at patients according to their specialty and not from a human perspective, mocking students, and not caring about student rounds.
Poor Clinical Competence	Lack of awareness of patients, undue insistence on diagnosis, failure to identify priorities, poor diagnosis, and weak clinical judgment and reasoning.
Insufficient Knowledge and Information	Inadequate knowledge, ignorance about scientific developments, outdated information, and inadequate familiarity with foreign languages.
Negative Personal Traits	Materialism and excessive regard for money, money worship, narcissism, bad temper, being too strict and inflexible, hypocrisy, being impolite, lack of confidence, being too proud, creating stress, low energy, selfishness, and lack of discipline.

The qualitative analysis showed that students had observed an absence of professional behaviors in some of their teachers. In this regard, a participant pointed out, *“I’ve witnessed several cases where the professor treated the students disrespectfully in front of the patients”*. Another participant said, *“I have seen professors who look at patients materialistically and not from a human perspective”*.

The results showed that students considered negative personal traits as a negative feature of role models. One participant stated, *“Some professors are unpredictable. You cannot tell whether they are in a good or bad mood. They are moody”*. Another student said, *“Some professors are arrogant and don’t look at the people they are talking to. They behave as if there is no one around”*. Another participant said, *“Some professors are used to insisting on their own views with no regard for others’ circumstances”*.

The participants considered insufficient knowledge and information as negative features of role models. One of them pointed out, *“In some classes, you notice the professor hasn’t updated himself/herself and just repeats what he/she has been teaching in the past years”*. 

The results showed that 46.8% of the students acknowledged one or more teachers as negative role models, whereas about 24.82% stated that there was no negative role model in the university, and about 28.36% did not respond to this question (Table 7).

**Table 7:** Students’ answers about negative role models in the university

Question 4	Positive Answers	Negative Answers	No Answers
Is there a teacher at the Shiraz University of medical sciences who has negative role model characteristics?	132 (28.36%)	70 (24.82%)	80 (28.36%)

## Discussion

Teachers play a vital role in medical education, and shape the students’ professional life and identity (14, 19, 27). In order to better understand and improve role modeling as an educational strategy, we need to have a clear view of the determining factors that cause a teacher to be seen as a role model. The results showed that students considered individual characteristics to be among the most important features in this regard. These results are in line with the findings of previous studies (1, 2, 12, 28, 29). For example, Wright and Carrese (2) showed that such features as interpersonal skills, positive views, commitment to promotion and development, and honesty could be effective in making a lecturer a role model. In general, it can be said that the individual characteristics of teachers can be powerful predictors for role modeling, and students pay special attention to their teachers’ characteristic traits. From the students’ perspective, teachers with the following personality traits were more interesting than others and had more positive effects: confidence, intelligence, the power to convey knowledge, piety, modesty, good manners, punctuality and the ability to adjust to the team; these teachers were also caring, helpful, committed and patient, authoritative, conscientious, sociable, creative and honest. Therefore, we can conclude that medical students are sensitive to their professors’ behaviors in all circumstances, and pay special attention to their conduct and characteristics. For that reason, professors should be aware of their behaviors and personality traits in educational environments, and constantly be careful about everything they say or do.

In line with previous research, the students in this study considered their teachers’ clinical skills and competence among the most significant aspects of role models (1, 2, 5, 12, 22, 24, 28- 31). In this respect, a study by Wright et al. (1) on the impact of role models on students showed that clinical competence is one of the significant factors in selection of role models.

It is the essence of medical education to ensure the best practices and to care for patients. The results showed that students considered the value of optimal patient care in the selection of these components as important factors in role modeling. Based on the students’ views, medical teachers made good role models if they had the following features: examined patient’s behavior thoroughly; had sound clinical judgment and reasoning, and excellent decision making abilities; had good patient management and diagnostic skills; could manage rounds efficiently; were cooperative; and had the capacity to supervise all aspects of treatment. 

The results also showed that teaching skills were regarded as the third important component of role models. This is in line with the findings of previous studies (1-3, 5, 22, 28, 31, 32), indicating that when professors improve their teaching skills, they will be more likely to be considered as positive role models. 

Another finding of our study demonstrated that professionalism could act as a critical component of role modeling. The results suggest that teachers with higher levels of professionalism are considered as positive role models by students, which has also been highlighted in previous studies (10, 23, 33). Some found that teachers’ professionalism can affect the students' selection of a teacher as a role model; hence, role modeling can be facilitated through interacting effectively, being compassionate toward others, behaving respectfully, practicing open and interactive communication, having a sense of humor, supporting the students fully and improving their self-esteem (23). In this regard, Reuler and Nardone (1994) concluded that self-criticism, responsibility, humility, and respect, sensitivity to patients and students are considered as characteristics of role models (34). 

The results of the qualitative analysis showed that the students’ views on the characteristics of positive role models could be classified into five main components including professionalism, clinical competence, teaching skills, updated knowledge and information, and individual characteristics. The qualitative results confirmed the quantitative results. In other words, the components and features of role models as extracted from the interviews were consistent with the results of the quantitative part of the study. The results also showed that 79% of the students reported at least one teacher at the university as a positive role model. In this regard, the results of Wright and Carrese’s study (2002) showed that 90% of the students identified a model or role model teacher during their studies at the school of medicine (2). Students observe their teachers’ behavior directly, and therefore, clinical teachers can improve their performance through awareness of their impact as role models. They should always be conscious and understand that all their interactions, personal views, behaviors and attitudes are observed and followed by students in various situations. 

Other results of the qualitative analysis showed that 46.8 percent of the students acknowledged one or more medical teachers as negative role models. Based on the students’ views, characteristics of a teacher who is a negative role model can be classified into four main components including unprofessional behavior, poor clinical competence, insufficient knowledge and information, and negative personal traits. In this regard, some studies have shown that more than 50 percent of the university faculty members were not seen as positive role models by the residents (14, 29). In addition, some researchers have pointed out the influence of negative role modeling in clinical settings (35, 36). For example, in a study conducted by Murakami et al. (2009), the result showed how the career choices and professional behaviors of students were adversely affected by negative role models (36). This issue occurs in the form of hidden curricula and in informal environments. Similarly, White et al. (2009) showed how students’ values and behaviors were shaped through the perceived conflicts between formal and informal curricula in medical schools. They showed that during clinical clerkships, medical students experienced serious inconsistencies between what they had read and learned regarding patient-centered care in the first two years and what they observed in their role models in the third year (35). Teachers have both positive and negative effects on the future professional behaviors of their students. There is a need for revision and consideration of all aspects of the learning environment, particularly the non-structured and hidden curricula, since these are the factors that determine students’ future professional behaviors. In other words, students are constantly observing and examining their professors' behaviors in all formal and informal environments, and are more affected by the latter. The results also showed that the students noted the professors’ negative roles in different circumstances. It is important to add that negative role models can have an equally strong influence on students' attitudes and professional behaviors, and students can easily imitate these behaviors. Considering the difficulty of controlling role models’ conduct, we should confirm the necessity of having a structured curriculum for teaching professional principles in medicine. Hence, educational planners and policymakers, as well as professors, should pay special attention to the role of hidden curricula and its effect on the future professional behavior of students. Finally, it is imperative that all clinical teachers be aware of their conduct and adopt proper techniques to reduce these negative effects. 

Generally, although some studies have been conducted on role modeling, the present article is one of the few articles using mixed method design which yields a better understanding of role modeling. Moreover, the other strength of this study is considering positive role models as well as negative ones mutually. This aspect of research will enrich the role modeling literature in the context of Iran and will provide a new insight as to role modeling in medical education.

## Conclusion

This study revealed that the characteristics of medical teachers as role models could be classified in the following domains: individual characteristics, clinical skills and competence, teaching skills and professionalism, in that order. Consequently, teachers' consciousness and self-awareness in education, personality, patient care and other behaviors are considered as the cornerstones of a good role model. Consciousness and self-awareness are the two most important characteristics of any teacher dedicated to presenting exemplary (praiseworthy) behaviors both inside and outside the classroom. Graduates’ future conduct will probably be dependent on these laudable behaviors in the classroom and outside. Hence, it is expected of teachers to exhibit behaviors that reflect moral virtues in compliance with professional conduct. 

## Suggestions

1. Encouraging and introducing positive role models and lecturers in the university to highlight appropriate conduct.

2. Sharing the results of this research with teachers in order to increase their awareness and reflect on the characteristics of positive and negative role models, as teachers’ awareness is the first step in improving their performance as role models. 

3. Inviting positive role models to present and share their experiences with other teachers. 

4. Granting privileges to or facilitating the promotion of professors who are considered as positive role models in order to encourage positive behaviors and examples.

5. Holding professional development programs for lecturers to improve their clinical skills, competence and teaching skills, as these are considered by students to be important features of role models. 

6. Holding self-reflection sessions for professors regarding their behaviors and individual characteristics such as piety and modesty, helpfulness, good manners, sociability and honesty. Our results showed that these factors are crucial in effective role modeling. 

## Citation to this article:

Bazrafkan L, Hayat AA, Tabei SZ, Amirsalari L. Clinical teachers as positive and negative role models: an explanatory sequential mixed method design. J Med Ethics Hist. 2019; 12: 11
